# High/low cortisol reactivity and food intake in people with obesity and healthy weight

**DOI:** 10.1038/s41398-020-0729-6

**Published:** 2020-01-27

**Authors:** Benedict Herhaus, Enrico Ullmann, George Chrousos, Katja Petrowski

**Affiliations:** 1 Medical Psychology and Medical Sociology, Clinic and Policlinic for Psychosomatic Medicine and Psychotherapy, University Medicine Mainz, Mainz, Germany; 2 Department of Medicine, Carl Gustav Carus, Technical University of Dresden, Dresden, Germany; 3 Department of Child and Adolescent Psychiatry, Psychotherapy, and Psychosomatics, University of Leipzig, Leipzig, Germany; 4 School of Medical Biology, South Ural State University, Chelyabinsk, Russia; 5 First Department of Pediatrics, Medical School, National and Kapodistrian University of Athens, Athens, Greece

**Keywords:** Human behaviour, Predictive markers

## Abstract

Increased food intake, termed “comfort eating”, is a pathologic coping mechanism in chronic stress. Cortisol reactivity under stress is a potent predictor of stress-induced eating behavior affecting the body mass index (BMI). However, cortisol reactivity and food intake under stress in people with obesity has not been evaluated. The aim of this study was to investigate the effect of high/low cortisol reactivity on food intake in people with obesity and healthy weight test controls, following standardized stress induction and a resting condition. Thirty-six men and women with obesity (BMI: 33.00 ± 3.23 kg/m²), as well as 36 age- and gender-matched healthy weight controls (BMI: 21.98 ± 1.81 kg/m²) were categorized into high cortisol reactors (HCR) and low cortisol reactors (LCR) in the Trier Social Stress Test (TSST). Following the TSST and a resting condition, the food intake of all participants was recorded in a standardized laboratory meal. Obese HCR demonstrated a significantly higher food intake than LCR (*t* (34) = −2.046, *p* ≤ 0.05). However, there were no significant differences between HCR and LCR in the healthy weight controls (*p* = 0.26). In addition, HCR of the people with obesity showed lower values in the emotion coping strategy of cognitive reappraisal than obese LCR (*t* (32) = 2.087, *p* ≤ 0.05). In conclusion, the magnitude of the cortisol reactivity to stress predicts stress-induced food intake in people with obesity, but not in the healthy weight controls. Limited use of cognitive reappraisal in emotion regulation in the obese HCR may be a marker of vulnerability to stress-induced eating.

## Introduction

The prevalence of obesity has doubled over the past 40 years, with ~20% incidence in European countries^1^. Eating behavior is a crucial factor in understanding the mechanisms of obesity development. Eating behavior is multifactorial, including food choices, meal times, and energy intake, while it is also influenced by personal and environmental factors^2^. Stress has a major impact both in people with cachexia- and obesity-related irregular eating patterns^3^.

### Stress and eating behavior

Previous studies on eating behavior in individuals with a healthy body weight showed that 70% of those interviewed reported an increase in food intake while faced with stress, while the remaining 30% reported a decrease in food intake during stress^4,5^. To investigate these diverging behaviors, laboratory settings were chosen. Two studies showed an increased amount in food consumption and preference of highly palatable food during stress^6,7^. In contrast, we and others demonstrated no changes in eating behavior or even a decrease in food intake during stressful periods^8–10^. These divergent findings might be explained by the complex interactions of physiological and psychological mechanisms, influencing stress-related eating behaviors^11^. Thus, psychological drivers, such as coping style, emotion regulation, and stress appraisal are important moderators of the association between stress and a change in eating behavior^11^. On the other hand, stress effects may lead to changes in the circulating concentrations of cortisol, insulin, ghrelin, and leptin, which may influence eating behavior^12–14^. As a possible psychophysiological mechanism of high/low glucocorticoid response to stress, the active offensive or passive defensive calming response mechanisms with different levels of physical activity were described in a chronic stress paradigm, regarding different allostatic set points^15^.

### Stress-induced eating in high/low cortisol reactors

Stress-induced eating and cortisol reactivity to an acute stressor might be related to eating behavior. Depending on their cortisol reactivity to a stressor, subjects may be divided into high and low reactors. The animal study by Hewagalamulage et al.^16^ showed that high cortisol reactors (HCR) demonstrated higher food intake and a reactive behavioral coping strategy during stress, in contrast to low cortisol reactors (LCR) who had a proactive behavioral coping strategy. Similarly, among lean women with a high cortisol reactivity in response to a laboratory stressor, there was an increased ad libitum food intake^6,17^. In naturalistic settings, HCR had a positive association of daily stress and increased snack intake, while no such relation was observed in LCR^18^.

### Interplay of cortisol reactivity, stress-induced eating, and obesity

It is striking how different eating disorders are associated with greater cortisol reactivity to acute laboratory challenges^19^. Diverse outcomes have been reported on the link between stress-induced cortisol reactivity and food intake in people with obesity. On the one hand, in women with obesity, higher cortisol reactivity in response to the Trier Social Stress Test (TSST) was associated with a reduced snack intake^12^. On the other hand, in a study by Geliebter et al.^20^, also in women with obesity, the cortisol reactivity to acute cold stress did not predict subsequent food intake. These divergent results might be explained by methodological differences, especially the nature of the stressor, between the studies, because of possible limitations regarding the lack of a control group with a low cortisol stress reaction^20^, and because only snacks were presented presenting as test food^12^.

### Objectives

This study, therefore, aimed to further investigate the effect of high and low cortisol stress reactivity on food intake, following an acute laboratory stress paradigm, and a resting condition in people with obesity and healthy weight controls. Based on the study by Epel et al.^6^, we hypothesized that healthy weight HCR would tend to eat more in response to acute stress than the low reactors (hypothesis 1). Given the greater cortisol reactivity of people with obesity^19^ and the effect of cortisol on food intake^21^, we hypothesized that obese HCR would show greater stress-induced food intake compared to low reactors (hypothesis 2). With regard to the link between basal cortisol level, cortisol reactivity, and eating behavior^6,22^, we hypothesized that healthy weight HCR would exhibit higher basal cortisol levels than the low reactors (hypothesis 3). In view of lower cortisol activity in obesity^23^, which might influence the stress-induced cortisol reactivity, we hypothesized that obese LCR would show lower basal cortisol levels compared to high reactors (hypothesis 4).

## Material and methods

### Study participants

Thirty-six men and women with obesity (body mass index, BMI: 33.00 ± 3.23 kg/m²) according to the International Classification of Diseases (ICD-10), as well as a group of 36 age- and gender-matched healthy weight controls (BMI: 21.98 ± 1.81 kg/m²) were recruited through newspaper advertisements, online tendering, and notice boards at different universities. Exclusion criteria (any acute and/or chronic medical illness, mental disorders, receiving medications, using substances, and having experienced stressful life events in the previous 6 months) were inquired and ascertained in a telephone interview based on the entire procedure of the Structured Clinical Interview (SCID)^24^ according the Diagnostic and Statistical Manual of Mental Disorders (DSM-IV)^25^. A detailed description of the subjects studied is given in Table 1. There were no significant differences between the two groups for age (*t* (70) = −1.757, *p* = 0.08) and gender (*χ*² = 0, *df* = 1, *p* = 1.00). All 72 participants received an allowance of 50 euros after successful participation. The study protocol was approved by the local Ethics Committee of the Medical Faculty of the Technical University of Dresden, Germany (No #EK46032008).

**Table 1:** Characteristics of the participants regarding matching criteria.

	People with obesity	Healthy weight controls	*t*/*x*²/*Z*	*p*
Total, *N*	36	36		
Females, *n* (%)	16 (44.4)	16 (44.4)		
Age, M (SD)	31.50 (8.16)	27.94 (8.99)	−1.757	0.08^b^
BMI, M (SD)	33.00 (3.23)	21.98 (1.81)	−17.837	≤0.001***^b^ (*d* = −4.26)
Smokers, *n* (%)	3 (8.3)	7 (19.4)	1.858	0.17^c^
Cigarettes/day (of smokers), M (SD)	11.33 (5.62)	4.53 (4.01)	−2.209	0.06^b^
Contraceptives *n* (% of females)	4 (25.0)	6 (43.8)	0.582	0.45^c^
TICS SCSS, M (SD)	17.20 (8.61)^a^	13.23 (7.19)^a^	−3.971	0.04*^b^
Medication intake, *n* (%)	0 (0.0)	0 (0.0)	0	1.000^c^
Sports activities per week in hh:mm, M (SD)	4:34 (3:37)	5:08 (2:52)	0.590	0.56^b^

*BMI* body mass index, *d* Cohen d; *M* mean; *SD* standard deviation; *TICS* Trier Inventory for Chronic Stress; *SCSS* Subscale of Chronic Stress.

*p* ≤ 0.05*; *p* ≤ 0.01**; *p* ≤ 0.001***.

^a^Sub-sample of *n* = 35 participants.

^b^Independent Student *t*-test.

^c^Chi-square test.

### Procedures

Two laboratory sessions (stress and resting) were completed over a time frame of 7 days. The testing sequence of the two conditions (TSST vs. resting condition) was randomized, but the time schedule was similar. No effect of starting condition (stress/rest) was found in the results (*F*(_1,70_) = 0.296, *p* ≤ 0.59). The 2-h-laboratory stress and resting session started between 2:00 p.m. and 4:00 p.m., taking in consideration the circadian rhythm of cortisol release. The participants were asked to refrain from eating for at least 3 h, and from drinking and smoking for at least 1 h before the testing session. There was no significant difference in the time period of the last meal before testing between both conditions (stress condition: mean = 239 ± 87 min; resting condition: mean = 247 ± 90 min; *t* (68) = −0.753; *p* = 0.45).

Both times, immediately upon their arrival at the laboratory, the participants were allowed a stationary period (~30 min), during which a clinical interview was taken and the Trier Inventory for Chronic Stress (TICS), as well as the Emotion Regulation Questionnaire (ERQ) were filled out. The experimental protocol started with a 15-min pre-session, in which two saliva samples were collected and two pieces of food were consumed while wearing a food intake sound sensor system. Afterward, the participants went through two 15-min periods (resting and stress condition). The TSST was performed according to the published process protocol by Kirschbaum et al.^26^, with three sections consisting of preparation, interview, and a calculation task (5 min for every block). During the resting condition, the participants were offered the opportunity to read magazines. To assess the cognitive appraisal during both condition periods, the questionnaire Primary Appraisal Secondary Appraisal (PASA) was filled out 3 min after the start of either condition. Also, saliva samples were collected 5 and 15 min after the start of the TSST and the resting condition. After the resting and stress periods, the participants evaluated their previous experience, as well as their appetite for food preferences, through the visual analog scale (VAS).

**Fig. 1:**
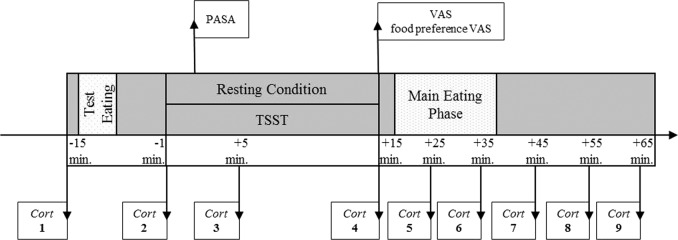
Procedure of laboratory sessions. Cort, salivary cortisol sample; PASA, Primary Appraisal Secondary Appraisal; TSST, Trier Social Stress Test; VAS, visual analog scale.

At the stress session, the participants were informed that a second stress test would be performed after the meal, in order to maintain a consistent feeling of stress during the eating period. For the test meal with one sweet and one non-sweet type of food, the participants were served four cheese sandwich halves (average weight of one half: 45 g ± 5 g), six cream-filled cookies (average weight of one biscuit: 13.8 g), and six dark chocolate biscuits (average weight of one biscuit: 14.0 g). In addition, the participants had the opportunity to drink an apple fizz drink (one bottle of 500 ml) and mineral water (one bottle of 500 ml). The participants were instructed by the investigator to “eat and drink as much as they would like”, while sitting at a table, and to let the investigator know when they were finished. At the stress session, the participants were informed after the eating period that the second stress test had been canceled so that they would be able to rest for the remaining time. During the 50-min post-session, five further saliva samples were taken at a time interval of 10 min each. For this purpose, the main eating phase was interrupted for cortisol samples for 1 min. A detailed description of the laboratory sessions is given in Fig. 1.

### Measures

#### Food intake and assessment

The laboratory foods and drinks were weighed before and after the study in a separate room using a kitchen scale (accuracy: 0.01 g), and their caloric values were calculated. The caloric content of the sandwich half was 3.8 kcal/g, while that of the full-cream milk/dark chocolate biscuit was 5 kcal/g. The general appetite (“How big is your general appetite at this moment?”) and the specific appetite for ten special food groups or foods (“How big is your appetite for the following food groups or foods at this moment?”) were assessed before the eating period via the nutritional preference VAS. These groups or foods are (1) sweet foods (e.g., cake, chocolate, biscuits, and ice cream), (2) fruits, (3) starchy foods (e.g., bread, noodles, muesli, and potatoes), (4) salty foods (e.g., chips, nuts, and olives), (5) vegetables, (6) meat, (7) milk foods (e.g., cheese and yogurt), (8) sour foods (e.g., sour gherkins), (9) fish, and (10) eggs. The VAS, which scores from 0 (no appetite) to 100 (extremely strong appetite), is a valid instrument frequently used in appetite research^27^. Furthermore, to collect influencing variables on the eating behavior, such as dental or chewing problems, as well as to control for the comprehension of the instructions, the participants filled out a health questionnaire.

#### Psychological assessments

The ERQ of Gross and John^28^ was used to assess the two regulatory strategies of expressive suppression and cognitive reappraisal. The questionnaire is based on ten items with a seven-point rating scale (1 “totally disagree” to 4 “neutral” to 7 “totally agree”) and evaluates the two scales of suppression (four items) and reappraisal (six items). Perceived chronic stress was measured by the TICS constructed by Schulz et al.^29^. This questionnaire retroactively evaluates nine interrelated factors and is based on 57 items (five-point rating scale). Finally, an additional screening subscale (SCSS) of psychosocial chronic stress during the previous 3 months was obtained. In the current sample, the internal consistency exhibited good reliability values of Cronbach’s *α* between 0.78 and 0.90 for the used scales of the ERQ and TICS.

The cognitive appraisal of the stress situation was measured by the PASA constructed by Gaab^30^ is based on 16 items (six-point rating scale) and measures the cognitive appraisal processes with the tertiary scale of “stress-index” (“primary appraisal” [(threat + challenge)/2] − “secondary appraisal” [(self-concept of own abilities + control expectancy)/2]. The reliabilities (Cronbach’s Alpha-coefficient) in the current sample of the two secondary scales (primary appraisal: 0.64; and secondary appraisal: 0.80) demonstrated reasonable reliability. The self-reported stress perception after the TSST was evaluated by the VAS. This test rates from 0 (no stress) to 100 (maximum stress) has previously proved to be valid^31^.

#### Cortisol samples

For the ascertainment of the cortisol concentrations, saliva samples were collected by moistening a cotton roll in the mouth for 1 min and then placing it into a salivette® (Sarstedt, Germany). The female participants completed the testing day in the luteal phase, in consideration of the influence of the menstrual cycle on cortisol^32^. The salivary cortisol concentrations were analyzed using a luminescence immunoassay test method. Based on the intra- and inter-assay coefficient of variation <9.0%^33^, this test method has proved to be robust and valid.

### Statistical analysis

In view of a statistically responsible sample, the optimum statistical sample size was calculated with the G*power program (version: 3.1.9.2.). To test hypothesis 1–4 (Introduction—objectives section) independent Student *t*-tests were performed. Based on an effect size of *d* = 0.80, a significant level of *p* = 0.05 and power of 80% (1 − *ß* = 0.80), a total sample size of *N* = 26 per subject group was needed for all four hypotheses. All statistical analyses were conducted using SPSS Statistics version 25 (IBM, Chicago, IL, USA). For a detailed analysis of the effect of the TSST on the cortisol concentration, the area under the curve with respect to increase (AUC_I_) was calculated^34^. For a specification of the cortisol reactivity to the stress condition, subgroups were formed separately for the two groups (people with obesity/healthy weight controls) by a median split of cortisol reactivity (AUC_I_) with regard to the TSST. Group-specific median split was performed with regard to equal sample size of high/low reactors in obese and healthy weight individuals, as well as the possible greater cortisol reactivity of people with obesity^19^. The 36 people with obesity were categorized into high (AUC_I_ > 4.211 nmol/l*min, *n* = 18) and low (AUC_I_ < 4.211 nmol/l*min, *n* = 18) reactor groups. The 36 healthy weight controls were also divided into high (AUC_I_ > 3.162 nmol/l*min, *n* = 18) and low (AUC_I_ < 3.162 nmol/l*min, *n* = 18) reactor groups. Compared to previous studies^6,18^, which performed a median split of the cortisol reactivity, the cutoffs for the two groups in the current study were higher.

For the evaluation of differences between the people with obesity and healthy weight controls in sociodemographic variables, perceived chronic stress, medication intake, and sports activities, the independent Student *t*-Test, Chi-square test, and Mann–Whitney *U* test were performed.

To analyze the effect of stress induction (stress vs. resting) on stress appraisal and hormonal response, analysis of variance (ANOVA) for repeated measurements was applied with the within-factor condition (stress vs. resting). Given that appetite may be an influencing factor on food intake, the appetite appraisal after both conditions was tested with the Wilcoxon signed-rank test for ordinal variables in all the participants.

The differences between the obese HCR and LCR, as well as between the healthy weight HCR and LCR in the parameters subjective appraisal (VAS, PASA, and ERQ), derived cortisol parameters (AUC_I_ and baseline level), as well as in the food intake (sandwich eaten, biscuit eaten, and total food intake) with regard to the resting condition and the stress condition were tested by independent Student t-test.

## Results

### Sample characteristics

A description of the 36 people with obesity (BMI: mean = 33.00 kg/m², SD = 3.23) and the 36 people with healthy weight (BMI: mean = 21.98 kg/m², SD = 1.81) is given in Table 1. There were no significant differences in the variables age (*t* (70) = −1.757, *p* = 0.08), use of oral contraceptives (*χ²* = 0.582, *df* = 1, *p* = 0.45), number of smokers (*χ²* = 1.858, *df* = 1, *p* = 0.17), and sports activities (*t* (70) = 0.590, *p* = 0.56). Concerning the perceived chronic stress, the people with obesity showed significantly higher values (*t* (68) = −3.971, *p* = 0.04) in the screening SCSS of the TICS (mean = 17.20, SD = 8.61) than the healthy weight controls (mean = 13.23, SD = 7.19).

### Stress induction and appetite appraisal

The anticipatory cognitive appraisal of stress (PASA) was significantly lower before the TSST than during the resting condition (*F*_(1, 70)_ = 145.943, *p* *≤* 0.001, *η*^2^ *=* 0.679). All the participants exhibited significantly higher values in the VAS after the TSST than during the resting condition (*F*_(1, 70)_ = 99.249, *p* *≤* 0.001, *η*^2^ *=* 0.586), which demonstrated perceived acute stress in the TSST. Furthermore, a significant effect of time with higher values in the stress condition in cortisol AUC_I_ (*F*_(1, 70)_ = 61.680, *p* *≤* 0.001, *η*^2^ *=* 0.468) was observed. This demonstrated a successful hormonal stress induction by the TSST. With regard to the appetite appraisal, there was significantly less general appetite after the stress condition, before the eating phase, than during the resting period (*z* = −2.610, *p* = 0.009) as well as less appetite for starchy foods (*z* = −2.161, *p* = 0.03), vegetables (*z* = −2.623, *p* = 0.009), fish (*z* = −2.898, *p* = 0.004), and eggs (*z* = −2.695, *p* = 0.007). More details of the stress induction and appetite appraisal can be found in Supplemental Tables 1 and 2.

### Derived cortisol parameters in HCR and LCR

As expected with regard to the median split of the cortisol reactivity in the groups of people with obesity and people with healthy weight, there were significant differences in the derived cortisol parameters AUC_I_ of the stress induction between the HCR and the LCR, but not in the resting condition. There were significantly lower baseline cortisol values in the obese LCR than the HCR on both conditions. The baseline cortisol levels in the healthy weight LCR and HCR revealed no significant difference before resting.

### Emotional regulation in HCR and LCR

Concerning emotional regulation, the obese HCR showed lower values in the subscale of cognitive reappraisal (mean = 3.67, SD = 1.45) than the obese LCR (mean = 4.55, SD = 0.98), with significant differences (*t* (32) = 2.087, *p* ≤ 0.05). In the suppression subscale, there were no significant differences between the two groups (*t* (32) = 1.117, *p* = 0.27); however, the obese HCR demonstrated lower values (mean = 3.06, SD = 1.16) than the obese LCR (mean = 3.51, SD = 1.20). In the group of the healthy weight controls, there were no significant differences (*p*’s > 0.07) between HCR and LCR in the subscale of cognitive new-appraisal (HCR: mean = 4.04, SD = 1.19; LCR: mean = 4.78, SD = 0.87) and suppression (HCR: mean = 3.61, SD = 1.01; LCR: mean = 2.92, SD = 1.62; Supplementary Table 1).

### Food intake in HCR and LCR

Table 2 demonstrates that only in the group of people with obesity there were significant differences in food intake parameters between the HCR and LCR groups. Therefore, the obese HCR showed a significantly higher intake after the stress condition in the parameters, regarding eaten sandwich (*t* (34) = −2.046, *p* ≤ 0.05) and total food intake (*t* (34) = −2.046, *p* ≤ 0.05) than the obese LCR. In the food intake parameters after the resting condition, there were no significant differences between the obese HCR and the LCR. In the group of the healthy weight controls, no significant differences were observed in the food intake parameters after stress or during the resting condition between the HCR and the LCR groups (*p*’s > 0.26).

**Table 2:** Hormonal reactivity and eating parameters in high/low reactors of people with obesity and healthy weight controls.

		People with obesity (*N* = 36)				Healthy weight controls (*N* = 36)			
		Low cortisol reactors (*n* = 18)	High cortisol reactors (*n* = 18)	Independent Student *t*-test		Low cortisol reactors (*n* = 18)	High cortisol reactors (*n* = 18)	Independent Student *t*-test	
Derived cortisol parameters		M (SD)	M (SD)	*t*	*p*	M (SD)	M (SD)	*t*	*p*
AUC_I_	Resting-C.	1.56 (53.66)	22.69 (113.07)	−0.716	0.48	3.24 (114.73)	17.98 (138.84)	−0.347	0.73
	Stress-C.	118.12 (100.34)	761.64 (336.28)	−7.780	≤0.001*** (*d* = −2.59)	67.71 (88.50)	762.21 (443.28)	−6.518	≤0.001*** (*d* = −2.17)
Baseline cortisol level	Resting-C.	2.29 (1.02)	6.36 (3.68)	−4.527	≤0.001*** (*d* = −1.51)	7.18 (3.91)	7.80 (4.43)	−0.453	0.65
	Stress-C.	2.70 (1.84)	5.56 (3.25)	−3.328	≤0.001*** (*d* = −1.08)	8.94 (4.23)	7.99 (4.26)	0.669	0.51
Eating parameters									
Eaten sandwich in kcal	Resting-C.	418.50 (236.45)	522.96 (301.25)	−1.157	0.26	563.93 (270.55)	577.18 (310.34)	−0.136	0.89
	Stress-C.	378.01 (240.65)	563.50 (299.95)	−2.046	0.05* (*d* = −0.68)	592.79 (348.25)	534.62 (278.12)	0.554	0.58
Eaten biscuit in kcal	Resting-C.	238.44 (250.82)	310.21 (154.67)	−1.033	0.31	249.67 (432.53)	202.44 (106.92)	0.450	0.66
	Stress-C.	208.39 (237.00)	290.88 (207.20)	−1.112	0.27	243.25 (270.40)	173.11 (105.75)	1.025	0.31
Total food intake in kcal	Resting-C.	656.95 (389.34)	833.17 (300.73)	−1.520	0.14	813.60 (511.70)	779.62 (365.25)	0.229	0.82
	Stress-C.	586.40 (424.08)	854.38 (359.03)	−2.046	0.05* (*d* = −0.68)	836.04 (350.52)	707.73 (324.10)	1.140	0.26

*AUC*_*1*_ area under the curve with respect to increase, *d* Cohen d, *kcal* kilocalories, *M* mean, *Resting-C.* resting condition, *SD* standard deviation, *Stress-C.* stress condition. *p* ≤ 0.05*; *p* ≤ 0.01**; *p* ≤ 0.001***

*p* ≤ 0.05*; *p* ≤ 0.01**; *p* ≤ 0.001***.

## Discussion

This study investigated the effect of high and low cortisol reactivity on food intake following a laboratory stress task (TSST) and a resting condition in people with obesity and healthy weight controls. As expected, the obese HCR demonstrated a significantly higher food intake after the TSST in contrast to the obese LCR. In contrast to our hypotheses, there were no significant differences between the healthy weight HCR and the LCR in the food intake after stress and the resting condition. Differences could be observed in the basal salivary cortisol levels between the obese HCR and LCR groups, but not between healthy weight HCR and LCR. With regard to the psychological drivers to stress-induced eating, the obese HCR showed lower emotion coping strategy of cognitive reappraisal than the obese LCR.

Several studies have demonstrated that heightened cortisol reactivity to stress is related to increased ad libitum food intake in healthy weight individuals^6,17,18^. In line with this, laboratory studies by Epel et al.^6^ and Newman et al.^18^, categorizing high and low cortisol reactor groups, showed an association between higher cortisol reactivity and increased food intake in individuals with healthy weight. The present study used a similar categorization of high and low reactor in people with obesity. The magnitude of the cortisol reactivity was connected to stress-induced food intake in people with obesity (BMI > 30) not in healthy weight individuals. In contrast, Appelhans et al.^12^ showed an association between higher cortisol reactivity and decreased snack intake in women with obesity, but not in women with healthy weight. Unfortunately, in the study by Appelhans et al.^12^, only snacks were offered as food choice (vs. a typical western lunch in this study), and there was no categorizing of high and low cortisol reactor groups with regard to the stressor.

Our data provide further support for a possible effect of cortisol reactivity on the food intake in people with obesity, but not in healthy weight controls. Therefore, the physiological mechanism of stress-induced eating is a complex interplay between many different hormones, whose secretion and activity are influenced by glucocorticoids^11,17^. It is still unclear why there is an effect of cortisol reactivity on food intake only in people with obesity and not in the healthy weight controls. One possible explanation might be a positive association between cortisol basal level and food intake. This is supported by the lower basal cortisol levels of the obese LCR than HCR. There is evidence in animal models, as well as in humans, that a high basal glucocorticoid level may lead to increased food consumption^22,35^, while a low basal glucocorticoid level also is associated with decreased food intake^36^. Due to the possible effects of basal cortisol levels on food intake, the study of Epel et al.^6^ demonstrated less food intake in LCR than HCR after stress induction, as well as lower basal cortisol in LCR than HCR.

A further possible explanation in view of the mechanism of stress-induced eating might be the alterations of the hypothalamic-pituitary-adrenocortical (HPA) axis in obesity, as the latter cortisol metabolism with regard to basal level and stress reactivity^19^. A disturbed feedback loop of the HPA axis may lead to resistance of adiposity signals in different body areas^21^. With regard to the selfish brain theory^37^, one possible mechanism might be altered signals from the HPA axis to the brain, resulting in failing regulatory processes of the energy balance and eating behavior. In addition, an HPA axis–leptin system imbalance was described in obesity^38^. The regulation of the neuroendocrine systems, including cortisol concentrations, might have modulatory roles of leptin action^39^. Further research should take into account the interplay between cortisol and the appetite-regulating hormones NPY, leptin, and ghrelin, by considering the potential delay of signaling pathways.

Stress cortisol reactivity might be involved in the physiological mechanism of stress-induced eating; however, enhanced cortisol reactivity might also be a marker of other psychological trait factors that influence stress-induced eating^6,17^. With regard to the effect of different eating styles, it must be considered that dietary restraint might be a risk factor for stress-induced overeating^40^. Also, there is an association between a high level of dietary restraint and enhanced cortisol reactivity^41^. Other studies also suggest that dysfunctional emotion regulation may lead to increased food intake^42–44^. Furthermore, there is evidence of an association between different body weight disorders and difficulties in emotional regulation^45^. As far as it concerns emotion regulation, the people with obesity categorized as HCR showed limited use of cognitive reappraisal; therefore, stress-related negative emotions were probably counteracted by eating a larger amount food via activation of the reward system. A limited use of emotional strategies leading to enhanced cortisol reactivity to an acute stressor has been previously reported^46^. Therefore, we suggest that obese individuals may be more vulnerable to developing stress-induced irregular eating patterns^47^. The strong association of obesity with preceding major life events^48^, suggests that many stressors in the life of people with obesity^49^, including their daily struggle with body weight loss and dieting, may lead to stress-induced eating as a pathologic emotional regulation coping strategy.

The strengths of this study are: the use of the standardized and reliable psychosocial stress test (TSST), the control of confounding factors of eating behavior (e.g., stress, appetite, and fasting state), inclusion of cortisol concentration measurements (e.g., circadian rhythm, baseline, and stress-induced), and two clearly separated groups according to ICD-10 into healthy weight controls (BMI ≤ 25 kg/m²) and people with obesity (BMI ≥ 30 kg/m²), with gender and age matching. The main limitation of this experimental study is the small sample size of *N* = 72, with only 36 people with obesity and 36 people with healthy weight, resulting in 18 participants in the HCR and the LCR in the two groups each. A factorial design with multivariate comparison (ANOVA) of the two conditions (resting and stress) and the four groups (healthy weight, obese, high/low reactors) would be more appropriate, but the sample size is too small to detect a two-way interaction. Despite the small sample size which, according to Robinson et al.^50^, is a problem in laboratory studies regarding eating behavior, significant effects were observed in our study. With regard to the food choice of one sweet and one non-sweet type of food, one might speculate that the food served have had an influence on the results of the food intake^6,51^. Another limitation is the non-assessment of other influential hormones with regard to the complex interplay of physiological mechanisms, resulting in stress-induced eating^51^. Finally, in order to standardize the circadian rhythm of the cortisol, study participants were investigated between 2:00 p.m. and 4:00 p.m., which is in the most middle European countries the time between the two meals lunch and tea time. It must considered, that this fact could influenced the results of the food intake.

The present data suggest that high cortisol stress reactivity might be a marker of vulnerability to stress-induced eating in obesity. In view of the increased prevalence of obesity and the impact of crucial irregular eating patterns, it is highly necessary to understand the role of cortisol in regulating appetite-related hormones and as to how alterations of the HPA axis are a dependent variable of stress-induced overeating. Furthermore, the results of this study indicate that improvement of the strategy of cognitive reappraisal might be an important factor in counteracting unhelpful stress-induced eating patterns and their effects on physiological and psychological pathways in people with obesity. Future research should concentrate on how treatments can effectively reduce stress-induced eating.

## Supplementary information

Supplemental Table 1

Supplemental Table 2

## References

[1] WHO. *Obesity and Overweight*. http://www.who.int/mediacentre/factsheets/fs311/en/ (2016).

[2] LaCaille L. In *Encyclopedia of Behavioral Medicine* (eds Gellman M. D. & Turner J. R.) 641–642 (Springer, New York, NY) 2013.

[3] Yau YHC, Potenza MN (2013). Stress and eating behaviors. Minerva Endocrinol..

[4] Oliver G, Wardle J (1999). Perceived effects of stress on food choice. Physiol. Behav..

[5] Kandiah J, Yake M, Willett H (2008). Effects of stress on eating practices among adults. Fam. Consum Sci. Res. J..

[6] Epel ES, Lapidus R, McEwen B, Brownell K (2001). Stress may add bite to appetite in women: a laboratory study of stress-induced cortisol and eating behavior. Psychoneuroendocrinology.

[7] Zellner DA (2006). Food selection changes under stress. Physiol. Behav..

[8] Kiessl GRR, Laessle RG (2016). Stress inhibits PYY secretion in obese and normal weight women. Eat. Weight Disord..

[9] Petrowski K, Wintermann G-B, Joraschky P, Päßler S (2014). Chewing after stress: psychosocial stress influences chewing frequency, chewing efficacy, and appetite. Psychoneuroendocrinology.

[10] Herhaus B, Päßler S, Petrowski K (2018). Stress-related laboratory eating behavior in adults with obesity and healthy weight. Physiol. Behav..

[11] Masih T, Dimmock JA, Epel E, Guelfi KJ (2017). Stress-induced eating and the relaxation response as a potential antidote: a review and hypothesis. Appetite.

[12] Appelhans BM, Pagoto SL, Peters EN, Spring BJ (2010). HPA axis response to stress predicts short-term snack intake in obese women. Appetite.

[13] Epel ES (2004). Are stress eaters at risk for the metabolic syndrome?. Ann. NY Acad. Sci..

[14] Rouach V (2007). The acute ghrelin response to a psychological stress challenge does not predict the post-stress urge to eat. Psychoneuroendocrinology.

[15] Ullmann E (2019). From allostatic load to allostatic state—an endogenous sympathetic strategy to deal with chronic anxiety and stress?. Front Behav. Neurosci..

[16] Hewagalamulage SD, Lee TK, Clarke IJ, Henry BA (2016). Stress, cortisol, and obesity: a role for cortisol responsiveness in identifying individuals prone to obesity. Domest. Anim. Endocrinol..

[17] George SA, Khan S, Briggs H, Abelson JL (2010). CRH-stimulated cortisol release and food intake in healthy, non-obese adults. Psychoneuroendocrinology.

[18] Newman E, O’Connor DB, Conner M (2007). Daily hassles and eating behaviour: the role of cortisol reactivity status. Psychoneuroendocrinology.

[19] Incollingo Rodriguez AC (2015). Hypothalamic-pituitary-adrenal axis dysregulation and cortisol activity in obesity: a systematic review. Psychoneuroendocrinology.

[20] Geliebter A (2012). Plasma cortisol levels in response to a cold pressor test did not predict appetite or ad libitum test meal intake in obese women. Appetite.

[21] Rutters F (2012). The hypothalamic-pituitary-adrenal axis, obesity, and chronic stress exposure: foods and HPA axis. Curr. Obes. Rep..

[22] Tataranni PA (1996). Effects of glucocorticoids on energy metabolism and food intake in humans. Am. J. Physiol..

[23] Champaneri S (2013). Diurnal salivary cortisol is associated with body mass index and waist circumference: the multi-ethnic study of atherosclerosis. Obesity.

[24] Wittchen HU, Zaudig M, Fydrich T (1997). Strukturiertes Klinisches Interview für DSM-IV..

[25] APA. *Diagnostic and Statistical Manual of Mental disorders, DSM-IV-TR*. 4th edn (American Psychiatric Association, Washington DC) 2000.

[26] Kirschbaum C, Pirke KM, Hellhammer DH (1993). The’Trier Social Stress Test’-a tool for investigating psychobiological stress responses in a laboratory setting. Neuropsychobiology.

[27] Flint A, Raben A, Blundell JE, Astrup A (2000). Reproducibility, power and validity of visual analogue scales in assessment of appetite sensations in single test meal studies. Int. J. Obes. Relat. Metab. Disord..

[28] Gross JJ, John OP (2003). Individual differences in two emotion regulation processes: implications for affect, relationships, and well-being. J. Pers. Soc. Psychol..

[29] Schulz P, Schlotz W, Becker P (2004). Trierer Inventar zum chronischen Stress (TICS)..

[30] Gaab J (2009). PASA - Primary appraisal secondary appraisal. Verhaltenstherapie.

[31] Lesage FX, Berjot S, Deschamps F (2012). Clinical stress assessment using a visual analogue scale. Occup. Med..

[32] Kudielka B. M., Hellhammer H. & Kirschbaum, C. In *Social Neuroscience: Integrating Biological and Psychological Explanations of Social Behavior* (eds Harmon-Jones E., Winkielman P.) 56–83 (Guilford Press, New York) 2007.

[33] Dressendörfer RA, Kirschbaum C, Rohde W, Stahl F, Strasburger CJ (1992). Synthesis of a cortisol-biotin conjugate and evaluation as a tracer in an immunoassay for salivary cortisol measurement. J. Steroid Biochem. Mol. Biol..

[34] Pruessner JC, Kirschbaum C, Meinlschmid G, Hellhammer DH (2003). Two formulas for computation of the area under the curve represent measures of total hormone concentration versus time-dependent change. Psychoneuroendocrinology.

[35] la Fleur SE, Akana SF, Manalo SL, Dallman MF (2004). Interaction between corticosterone and insulin in obesity: regulation of lard intake and fat stores. Endocrinology.

[36] Laugero KD (2001). A new perspective on glucocorticoid feedback: relation to stress, carbohydrate feeding and feeling better. J. Neuroendocrinol..

[37] Peters A (2004). The selfish brain: competition for energy resources. Neurosci. Biobehav. Rev..

[38] Morrison CD (2008). Leptin resistance and the response to positive energy balance. Physiol. Behav..

[39] Ahima RS (1996). Role of leptin in the neuroendocrine response to fasting. Nature.

[40] Wallis DJ, Hetherington MM (2004). Stress and eating: the effects of ego-threat and cognitive demand on food intake in restrained and emotional eaters. Appetite.

[41] Rutters F, Nieuwenhuizen AG, Lemmens SGT, Born JM, Westerterp-Plantenga MS (2009). Hyperactivity of the HPA axis is related to dietary restraint in normal weight women. Physiol. Behav..

[42] Evers C, Marijn Stok F, de Ridder DTD (2010). Feeding your feelings: emotion regulation strategies and emotional eating. Personal. Soc. Psychol. Bull..

[43] Metcalfe J, Mischel W (1999). A hot/cool-system analysis of delay of gratification: dynamics of willpower. Psychol. Rev..

[44] Telch CF, Agras WS (1996). Do emotional states influence binge eating in the obese?. Int. J. Eat. Disord..

[45] Cardi V, Leppanen J, Treasure J (2015). The effects of negative and positive mood induction on eating behaviour: a meta-analysis of laboratory studies in the healthy population and eating and weight disorders. Neurosci. Biobehav. Rev..

[46] Lam S, Dickerson SS, Zoccola PM, Zaldivar F (2009). Emotion regulation and cortisol reactivity to a social-evaluative speech task. Psychoneuroendocrinology.

[47] Sinha R, Jastreboff AM (2013). Stress as a common risk factor for obesity and addiction. Biol. Psychiatry.

[48] Pasco JA, Williams LJ, Jacka FN, Brennan SL, Berk M (2013). Obesity and the relationship with positive and negative affect. Aust. N. Zeal. J. Psychiatry.

[49] Abraham SB, Rubino D, Sinaii N, Ramsey S, Nieman LK (2013). Cortisol, obesity, and the metabolic syndrome: a cross-sectional study of obese subjects and review of the literature. Obesity.

[50] Robinson E, Bevelander KE, Field M, Jones A (2018). Reprint of ‘Methodological and reporting quality in laboratory studies of human eating behavior’. Appetite.

[51] Lemmens SG, Rutters F, Born JM, Westerterp-Plantenga MS (2011). Stress augments food ‘wanting’ and energy intake in visceral overweight subjects in the absence of hunger. Physiol. Behav..

