# Supplementary material for: High/low cortisol reactivity and food intake in people with obesity and healthy weight
Source: Transl Psychiatry. 2020 Jan 27;10:40. doi: 10.1038/s41398-020-0729-6 (PMC7026436; doi:10.1038/s41398-020-0729-6)
Supplement: Supplementary file 1 — Supplemental Table 1 [file 41398_2020_729_MOESM1_ESM.docx]

**Supplemental Table 1.**

Subjective appraisal in high/low reactors of people with obesity and healthy weight controls

|  |  | | **People with obesity (N=36)** | |  | | **Healthy weight controls (N=36)** | |  | |
| --- | --- | --- | --- | --- | --- | --- | --- | --- | --- | --- |
|  |  | | Low cortisol reactors (n=18) | High cortisol reactors (n=18) | Independent Student t-test | | Low cortisol reactors (n=18) | High cortisol reactors (n=18) | Independent Student t-test | |
| **Subjective Appraisal** | | | M (SD) | M (SD) | *t* | *p* | M (SD) | M (SD) | *t* | *p* |
| PASA - Stress index | | Resting-C. | -1.71 (.88) | -2.59 (1.45) | 2.208 | **.034***  *(d* = -.73) | -2.57 (.85) | -2.23 (1.06) | -1.065 | .29 |
|  |  | Stress-C. | -.42 (1.06) | -.35 (1.27)^a)^ | -.179 | .86 | -.76 (.96) | -.56 (1.05) | -.580 | .57 |
| VAS | | Resting-C. | 37.59 (15.06) | 41.70 (7.78) | -1.028 | .31 | 33.47 (10.77) | 37.86 (9.23) | -1.312 | .20 |
|  |  | Stress-C. | 59.24 (14.60) | 53.79 (14.66) | 1.117 | .27 | 55.65 (13.17) | 53.53 (6.36) | -.612 | .54 |
| ERQ cognitive re-appraisal | | | 4.54 (.98) | 3.67 (1.45)^a)^ | -2.087 | **.045***  *(d* =.70) | 4.78 (.87) | 4.04 (1.19)^a)^ | 1.874 | .07 |
| ERQ suppression | | | 3.51 (1.19) | 3.06 (1.15)^a)^ | 1.117 | .27 | 2.92 (1.62) | 3.61 (1.01)^a)^ | -1.398 | .17 |

Note. d = Cohen d; ERQ = Emotional Regulation Questionnaire; kcal = Kilocalories; M = Mean; PASA = Primary Appraisal Secondary Appraisal; Resting-C. = Resting condition; SD = Standard deviation; Stress-C. = Stress condition; VAS = Visual Analogue Scale; a) Sub-sample; p ≤ .05*; p ≤ .01**; p ≤ .001***
