# Supplementary material for: High/low cortisol reactivity and food intake in people with obesity and healthy weight
Source: Transl Psychiatry. 2020 Jan 27;10:40. doi: 10.1038/s41398-020-0729-6 (PMC7026436; doi:10.1038/s41398-020-0729-6)
Supplement: Supplementary file 2 — Supplemental Table 2 [file 41398_2020_729_MOESM2_ESM.docx]

**Supplemental Table 2.**

Appraisal of appetite in high/low reactors of people with obesity and healthy weight controls

|  |  | | **People with obesity (N=36)** | |  | | **Healthy weight controls (N=36)** | |  | |
| --- | --- | --- | --- | --- | --- | --- | --- | --- | --- | --- |
|  |  | | Low cortisol reactors (n=18) | High cortisol reactors (n=18) | Mann-Whitney-U test | | Low cortisol reactors (n=18) | High cortisol reactors (n=18) | Mann-Whitney-U test | |
| **Appraisal of Appetite** | | | Median (25-75% percentile) | Median (25-75% percentile) | *Z* | *p* | Median (25-75% percentile) | Median (25-75% percentile) | *Z* | *p* |
| Total appetite | | Resting-C. | 64.00 (42-77) | 70.00 (54-78) | -1.076 | .29 | 70.00 (57-76) | 68.00 (34-76)^a)^ | -.662 | .53 |
|  |  | Stress-C. | 55.25 (31-76) | 61.00 (35-71) | -.127 | .91 | 52.00 (41-75) | 50.00 (24-69)^a)^ | -.727 | .48 |
| Preference for food groups or foods | | |  |  |  |  |  |  |  |  |
| Sweet | | Resting-C. | 43.00 (27-68) | 70.00 (54-78) | -.348 | .74 | 63.00 (29-75) | 47.50 (14-81)^a)^ | -.116 | .91 |
|  |  | Stress-C. | 58.50 (10-70) | 61.00 (35-71) | -.427 | .67 | 38.50 (20-62) | 45.00 (18-63)^a)^ | -.099 | .94 |
| Fruits | | Resting-C. | 45.00 (14-74) | 29.50 (19-71) | -.332 | .74 | 50.00 (33-73) | 50.00 (23-70)^a)^ | -.281 | .78 |
|  |  | Stress-C. | 49.00 (10-73) | 37.00 (15-77) | -.981 | .34 | 64.00 (22-76) | 40.00 (22-66)^a)^ | -.578 | .57 |
| Starchy foods | | Resting-C. | 59.00 (19-79) | 47.50 (29-59) | -.206 | .84 | 50.00 (38-70) | 50.00 (18-74)^a)^ | -.496 | .64 |
|  |  | Stress-C. | 40.50 (19-68) | 36.00 (16-59) | -.063 | .96 | 50.00 (25-70) | 43.00 (13-63)^a)^ | -.712 | .48 |
| Salty | | Resting-C. | 34.50 (7-62) | 62.50 (37-76) | -.538 | .61 | 40.00 (10-52) | 29.00 (7-38)^a)^ | -.942 | .35 |
|  |  | Stress-C. | 40.50 (19-68) | 50.50 (23-66) | -.554 | .58 | 28.00 (7-55) | 20.00 (10-36)^a)^ | -.546 | .59 |
| Vegetables | | Resting-C. | 57.50 (20-69) | 29.00 (10-50) | -.839 | .41 | 45.00 (35-55) | 46.50 (22-66)^a)^ | -.182 | .86 |
|  |  | Stress-C. | 25.50 (9-69) | 31.50 (2-46) | -.285 | .78 | 50.00 (32-62) | 35.00 (20-58)^a)^ | -.829 | .39 |
| Meat | | Resting-C. | 51.50 (17-72) | 48.00 (41-75) | -.665 | .52 | 55.00 (27-68) | 13.50 (5-41)^a)^ | -2.795 | .004** |
|  |  | Stress-C. | 42.00 (11-67) | 28.00 (13-54) | -.760 | .45 | 42.50 (5-63) | 20.00 (1-67)^a)^ | -1.210 | .23 |
| Milk foods | | Resting-C. | 54.50 (16-69) | 69.50 (15-81) | -.775 | .44 | 65.00 (58-70) | 40.00 (20-65)^a)^ | -2.337 | .019* |
|  |  | Stress-C. | 31.50 (18-76) | 44.00 (9-75) | -.744 | .46 | 64.50 (33-70) | 50.00 (24-73)^a)^ | -.529 | .61 |
| Sour foods | | Resting-C. | 18.50 (3-56) | 62.50 (15-81) | -.951 | .36 | 25.00 (10-44) | 13.00 (4-45)^a)^ | -1.042 | .30 |
|  |  | Stress-C. | 13.00 (5-38) | 33.00 (10-61) | -.951 | .36 | 27.50 (5-50) | 15.00 (8-38)^a)^ | -.861 | .41 |
| Fish | | Resting-C. | 44.50 (7-69) | 10.00 (3-32) | -1.157 | .25 | 45.00 (15-52) | 14.00 (4-28)^a)^ | -1.821 | .07 |
|  |  | Stress-C. | 18.00 (7-35) | 12.00 (4-19) | -.808 | .42 | 23.00 (2-46) | 20.00 (4-45)^a)^ | -.100 | .92 |
| Eggs | | Resting-C. | 57.00 (6-72) | 25.00 (1-59) | -.918 | .37 | 35.00 (8-55) | 20.00 (13-41)^a)^ | -1.009 | .32 |
|  |  | Stress-C. | 29.50 (6-52) | 13.00 (1-31) | -.079 | .94 | 32.00 (9-41) | 15.00(6-28)^a)^ | -1.372 | .17 |

Note. Resting-C. = Resting condition; Stress-C. = Stress condition; a) Sub-sample; p ≤ .05*; p ≤ .01**; p ≤ .001***
